# Comparative Cytogenetic Study of Eggplant (*Solanum melongena* L.) and Its Wild Ancestors *Solanum insanum* L. and *Solanum incanum* L.

**DOI:** 10.3390/plants15162450

**Published:** 2026-08-12

**Authors:** Egizia Falistocco, Marilena Ceccarelli

**Affiliations:** 1 Department of Agricultural, Food and Environmental Sciences, University of Perugia, 06100 Perugia, Italy; 2 Department of Chemistry, Biology and Biotechnology, University of Perugia, 06123 Perugia, Italy; marilena.ceccarelli@unipg.it

**Keywords:** *Solanum melongena* L., *Solanum insanum* L., *Solanum incanum* L., karyotype, FISH, GISH, rDNA, repetitive DNA, domestication

## Abstract

Eggplant (*Solanum melongena* L., 2*n* = 2*x* = 24) is one of the most economically important crop species of the Solanaceae family. In recent decades, various approaches have enabled the identification of *Solanum insanum* as the direct ancestor and *Solanum incanum* as the closest wild relative; however, these studies have largely neglected chromosomal features. To expand the chromosome knowledge of eggplant and its ancestors, we performed a comparative cytogenetic study by combining karyomorphological analyses with Fluorescence In Situ Hybridization (FISH) and Genomic In Situ Hybridization (GISH). The three species exhibited similar chromosome morphology, except for the NOR-bearing chromosomes, which distinguished the *S. incanum* karyotype from those of the other two species. rDNA FISH mapping revealed two distinct patterns: one common to eggplant and *S. insanum*, and the other exclusive to *S. incanum*. The s-GISH method, used to examine the chromosome distribution of satDNA repeats, revealed identical patterns, which suggests that the chromosome structure of the investigated species was conserved during their evolution. Hybridization signals from cross-GISH experiments aligned with s-GISH patterns and confirmed the genomic homology among *S. melongena*, *S. insanum* and *S. incanum*. Our study provided novel cytogenetic evidence clarifying the evolutionary relationships between the cultivated eggplant and its ancestors.

## 1. Introduction

Solanaceae is one of the most important angiosperm families supplying humans with food, drugs and ornamental plants. It comprises approximately 3000 species classified into about 90 genera. Among these, *Solanum* L. is the largest and economically most important genus, comprising 1245 currently recognized species. It includes widely distributed crops such as potato (*S. tuberosum* L.), tomato (*S. lycopersicum* L.), and eggplant (*S. melongena* L.), alongside numerous minor crops cultivated in localized regions. Besides tomato, eggplant is the most economically important fruit crop of Solanaceae [1,2]. The name “eggplant” first appeared in 1763 and was originally applied to cultivars with white fruits that closely resembled chicken eggs [3]. In addition to the popular *S. melongena*, two underutilized African species, the scarlet eggplant (*Solanum aethiopicum* L.) and the gboma eggplant (*Solanum macrocarpon* L.), have considerable local importance in sub-Saharan Africa [1,2,4].

Eggplant has a long history of cultivation, and different approaches have been applied to shed light on its origin, wild relatives and domestication [2,5,6]. The migration of *Solanum incanum* L. from its native area, Northern Africa, to tropical Asia is supposed to have given origin to *Solanum insanum* L., which is unanimously accepted as the direct wild progenitor of the eggplant [2,4,7,8]. The geographical origin of the domestic eggplant has long been debated. According to Vavilov [9], *S. melongena* is native to the “Indo-Chinese center of origin”. Archaeological finds suggest that the utilization of the wild eggplant may have begun in India, with a subsequent additional and independent center of domestication in the Philippines [1]. Around the 10th century, the cultivated eggplant was introduced from Asia to North Africa and the Iberian Peninsula. Soon after the arrival of Europeans, it was introduced to America and expanded to other parts of the world [10].

*Solanum melongena* is highly nutritious due to its low calorie value and high content of vitamins, minerals, and bioactive compounds [11,12,13,14]. Human selection, along with mutation and hybridization, has contributed to the remarkable diversity of today’s eggplants. Cultivars vary significantly in their morphology (fruit size, color, shape, hairiness, and thorniness), physiology (flowering time and water requirements), and biochemistry (fruit bitterness) [15,16]. Eggplant is a diploid species with a genome size of approximately 1.17 Gb [17,18] and a chromosome number 2*n* = 24 [19], the same as its direct wild ancestor *S. insanum* and its close relative *S. incanum*. Polyploid variants have never been found. This is consistent with the scarcity of polyploidization events in the evolutionary history of the Solanaceae family [19]. Our knowledge of the eggplant genome has increased enormously in recent decades due to the continuous improvement of genome sequencing technologies. Since 2014, when the first draft of a reference genome was released [20], several improved genome sequences of the cultivated eggplant have been published [17,21,22,23,24,25]. Recently, a complete gap-free telomere-to-telomere genome assembly was achieved, and the physical map was successfully integrated with a cytogenetic map [18].

Specific cytogenetic investigations of the eggplant and its ancestors have never been performed. To our knowledge, chromosome studies on *S. insanum* are limited to karyological analyses of conventionally stained mitotic metaphases [26], whereas for *S. incanum*, only the chromosome number is known [7].

In this study, we aimed to advance the cytogenetic knowledge of *S. melongena* and its closest wild relatives, *S. insanum* and *S. incanum*. By integrating karyomorphological analysis with FISH (Fluorescence In Situ Hybridization) and GISH (Genomic In Situ Hybridization) techniques, we produced robust data to evaluate the cytogenetic affinities and evolutionary relationships among these species.

The FISH procedure was used to localize the chromosome sites of the rRNA genes (35S rDNA and 5S rDNA), which, due to their ubiquity in eukaryotic genomes, are considered excellent markers for the cytogenetic characterization of species and genera. 35S rDNA, also referred to as 45S rDNA in some literature [27], consists of three coding genes (18S, 5.8S, and 26S) organized in tandem repeats that form the nucleolar organizer regions (NORs) typically visualized as secondary constrictions. Also, 5S rDNA genes are present as multiple copies arranged in tandem arrays. Usually, the two clusters are organized in distinct sites located in different chromosomes.

For a more complete knowledge of the karyotype structure of these species, we applied a variant of GISH, known as self-GISH (s-GISH). This technique, consisting of the hybridization of the genomic DNA of a species to its own chromosomes, is a very helpful tool to localize the repetitive DNA on plant chromosomes, thus providing cytogenetic information on the prevalence of dispersed or tandem repeats, such as satellite DNA (satDNA). Finally, cross-GISH experiments were carried out to evaluate the genomic affinity between the cultivated eggplant and its ancestors, as well as between the ancestors themselves.

## 2. Materials and Methods

### 2.1. Plant Material, DNA Extraction and Probe Preparation

Eggplant varieties are of commercial source, the seeds were provided by a specialized nursery of Perugia (Italy). Several cultivars were investigated to identify potential intraspecific variation. Seeds of *S. insanum* and *S. incanum* were provided by Dr J. Prohens (COMAV, Instituto de Conservaciòn y Mejora de la Agrodiversidad Valenciana, Universidad Politécnica de Valencia, Valencia, Spain). The list of cultivars and accessions studied here is reported in Table 1.

Seeds were germinated according to the protocol of Ranil et al. [28]. The resulting seedlings were planted in pots to grow plants and obtain roots and leaves. Total genomic DNA used for the GISH probes was extracted from young, newly formed leaves. Extraction was performed using the DNeasy Plant Pro Kit (Qiagen, Hilden, Germany) according to the manufacturer’s instructions. The probe used to identify the 35S rDNA sites was the clone pTa71 containing the 18S-5.8S-25S rRNA genes and non-transcribed spacers of *Triticum aestivum* L. [29]. For the detection of 5S rDNA sites, the clone pXVI comprising the complete gene of 5S rRNA and the spacer region of *Beta vulgaris* L. [30] was used. The probes were labeled by nick translation, clone pTa71 with digoxigenin-11-dUTP (Roche, Merck KGaG, Darmstadt, Germany) and clone pXVI with biotin-14-dATP (Thermo Fisher Scientific Inc., Waltham, MA, USA). For the GISH experiments, total genomic DNA of each species was labeled with biotin-14-dATP (Thermo Fisher Scientific Inc., USA) by nick translation.

### 2.2. Chromosome Preparations

To accumulate metaphases, actively growing root tips 5–7 mm long were collected from plants and treated with ice-cold water for 24 h at 4 °C. Then, they were immersed in a 2 mM 8-hydroxyquinoline solution for 5 h at room temperature and fixed in 3:1 absolute ethanol-glacial acetic acid for at least 24 h at room temperature. Chromosome preparations were obtained using both dropping and squashing techniques. For the dropping method, fixed root tips were washed in enzyme buffer (10 mM citric acid/sodium citrate, pH 4.6) for 20 min and then transferred to the enzyme mixture containing 4% cellulase Onozuka R10 and 1% pectolyase (Sigma-Aldrich, Co., Steinheim, Germany) for 1 h at 37 °C. The cell suspension was pelleted and resuspended in the enzyme buffer. After pelleting, the material was washed twice with the fixative and resuspended in the fixative. Finally, 10–20 µL of cell suspension was dropped onto each slide. The slides were air-dried, observed under a phase contrast microscope to select the best preparations and then stored at −20 °C until used. Squashing was realized by using root tips digested as above and transferred onto slides. Meristematic tissue was dissected and squashed under a coverslip in a drop of 60% acetic acid. After removing coverslips by freezing at −80 °C, slides were air-dried, selected and stored as above.

### 2.3. Chromosome Counts and Karyotype Analysis

For each accession, 10 to 15 plants were used for this study. Karyotype analysis was performed at a minimum of ten mitotic metaphases per accession, showing a similar degree of chromosome contraction. The centromeric position was calculated as the long: short arm ratio for the classification of chromosomes according to the system of Levan et al. [31], that is, m = metacentric (r = 1.00–1.69), sm = submetacentric (r = 1.70–2.99), and st = subtelocentric (r = 3.00–6.99). The chromosome length was measured using ImageJ software v. 1.53 [32,33].

### 2.4. FISH and GISH Experiments

For in situ hybridization experiments, the slides were pretreated with 100 μg/mL of RNase A in 2 × SSC and then incubated in 40 units/mL of Pepsin (Sigma-Aldrich) for 15 min at 37 °C. The hybridization mixture consisted of 2 ng/μL of ribosomal probe and 5 ng/μL of genomic probe, 50% (*v*/*v*) formamide, 10% (*w*/*v*) dextran sulfate, 0.1% (*w*/*v*) SDS (sodium dodecyl sulfate) and 300 ng/μL sheared salmon sperm DNA. This last component was not included in the mix for GISH. After incubation for 10 min at 70 °C, the mixture was applied to the slides and denatured together with the chromosome preparations at 70 °C for 5 min and allowed to hybridize overnight at 37 °C. After hybridization, the slides were washed twice in 20% formamide (*v*/*v*) in 0.1 × SSC, for 5 min each. Detection of the digoxigenin- and biotin-labeled probes was carried out with anti-digoxigenin conjugated with FITC (Roche) and streptavidin conjugated with Cy3 (Sigma-Aldrich), respectively. The slides were counterstained with 2 μg/mL of DAPI (4′,6-diamidino-2-phenylindole) and mounted in antifade solution CitiFluor^TM^ AF1 (Electron Microscopy Sciences, Hatfield, PA, USA). The slides were examined with a Leica DMRB epifluorescence microscope (Leica Microsystems, Wetzlar, Germany). Images were captured with a digital photocamera SONY ILCE-7 (Sony Corporation, Tokyo, Japan) and then processed using Adobe Photoshop 5.0.

## 3. Results

### 3.1. Chromosome Numbers and Karyotype Features

**Figure 1:**
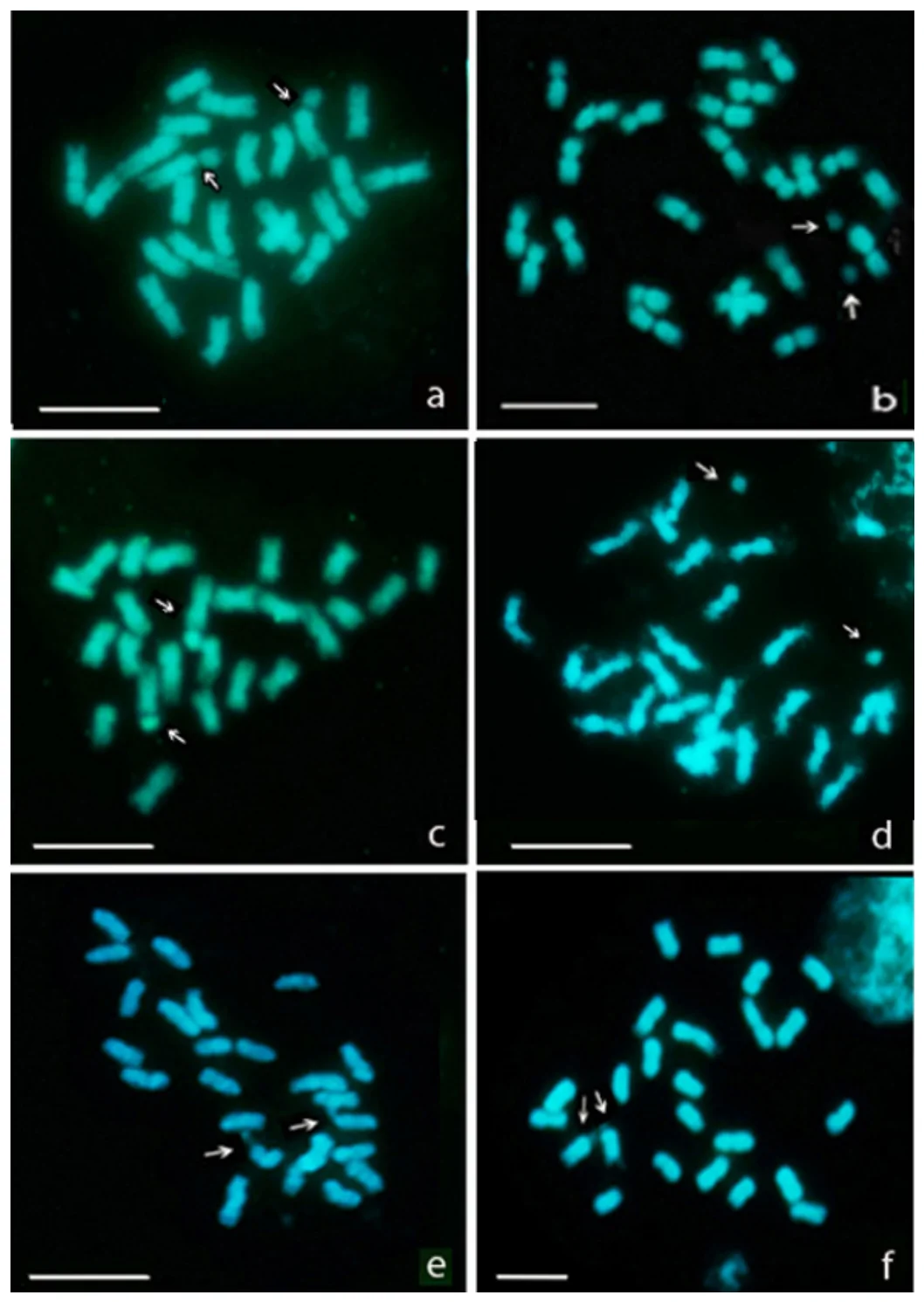
Mitotic metaphases of the *Solanum* species studied after DAPI staining. (**a**,**b**) Metaphase chromosome spreads of *S. melongena*. Arrows indicate the nucleolar chromosome pair with secondary constrictions and satellites (**a**) and the satellites apparently detached from their respective chromosomes (**b**). (**c**,**d**) Metaphases of *S. insanum*. Arrows indicate the nucleolar chromosome pair with secondary constrictions and satellites (**c**) and the satellites apparently detached from their respective chromosomes (**d**). (**e**,**f**) Metaphases of *S. incanum*. Arrows indicate slightly decondensed secondary constrictions (**e**) and condensed secondary constrictions (**f**). Bar represents 10 µm.

We determined a chromosome number of 2*n* = 24 in each eggplant cultivar and in the studied accessions of *S. insanum* and *S. incanum*, thereby confirming the typical chromosome number of these species (Figure 1). The chromosome analysis of DAPI-stained metaphases revealed a close similarity among the cultivars of *S. melongena.* Consequently, three metaphases for each cultivar were selected to define the karyotype of this species. It includes metacentric chromosomes with an arm ratio from 1.0 to 1.6, the length of which gradually decreased from the largest (6.1 μm) to the smallest (4.5 μm) chromosomes. One chromosome pair bearing a secondary constriction and a large satellite localized in the short arm was clearly detected in some metaphases (Figure 1a). However, due to the decondensation of the NORs, the satellites often appeared distant from their respective chromosomes, which were, therefore, difficult to identify (Figure 1b). Satellites typically exhibited weak DAPI staining; in some instances, they remained completely unstained and consequently undetectable.

The karyomorphological traits of *S. insanum* were found to be very similar to those of *S. melongena*. All chromosomes are metacentric, with an arm ratio from 1.2 to 1.5 and a length between 6.0 and 4.2 μm. A pair of NOR-bearing chromosomes similar to those found in eggplant cultivars was observed, notably showing terminal secondary constrictions, and weakly stained satellites were often dispersed and had no evident connection to their chromosome (Figure 1c,d).

The karyotype of *S. incanum* was determined here for the first time. It consists of metacentric chromosomes with an arm ratio from 1.3 to 1.6 and a length ranging from 5.9 to 4.3 μm. NORs are terminally located on the short arm of a chromosome pair, appearing as secondary constrictions, generally slightly decondensed or condensed, and not associated with the satellite-like structures, as observed in eggplant and *S. insanum* (Figure 1e,f).

Based on chromosome analysis, the three karyotypes may be classified as symmetric and defined by the formula 2*n* = 2*x* = 24: 24 m. The structure of the nucleolar chromosomes is the only karyological trait distinguishing *S. incanum* from *S. melongena* and *S. insanum*.

### 3.2. Chromosome Localization of the rDNA Sites by FISH

**Figure 2:**
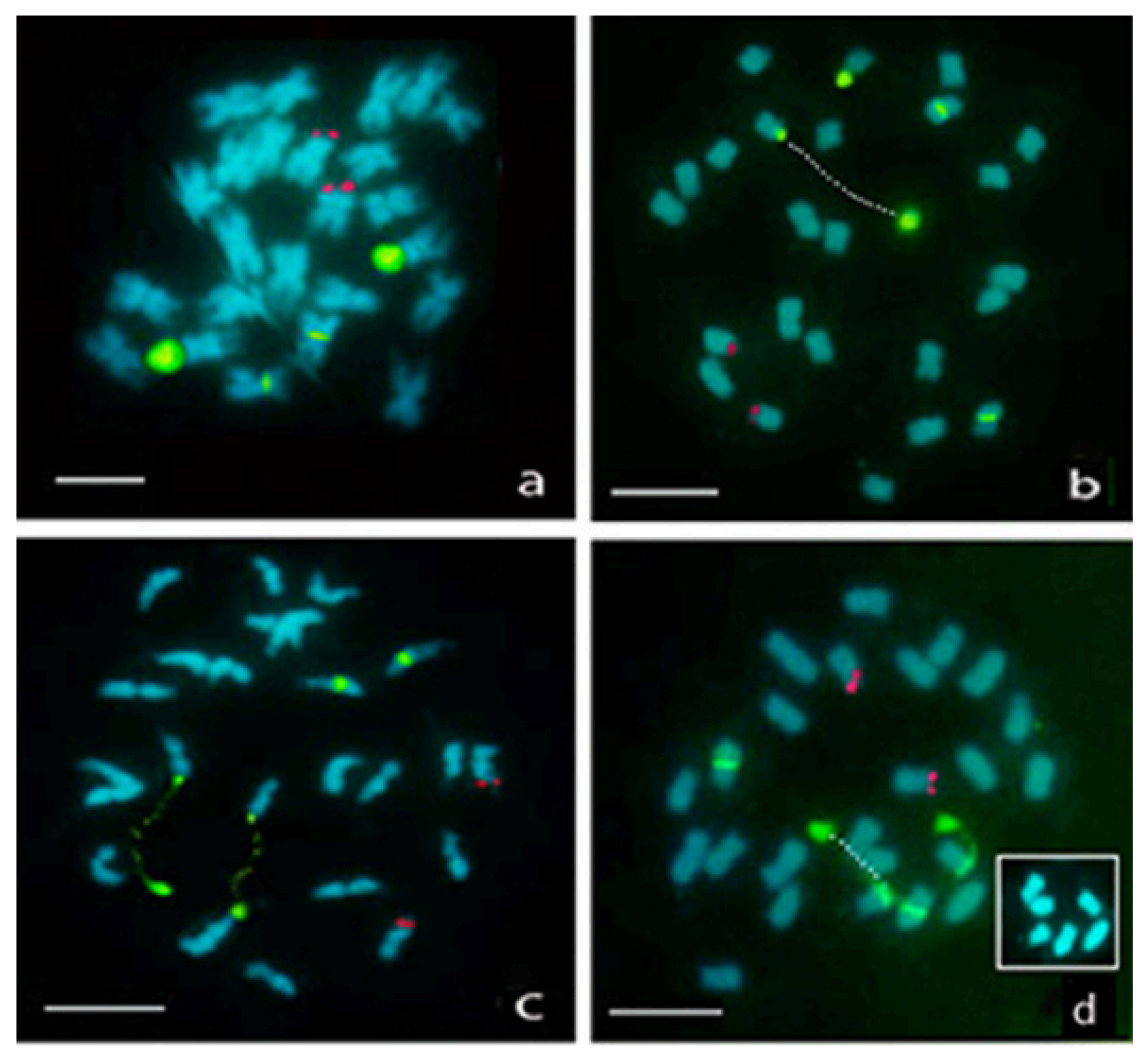
Fluorescent in situ hybridization with 35S (green) and 5S (red) rDNA probes in *S. melongena*. (**a**) Two large green signals correspond to the condensed NOR regions. (**b**) One NOR is highly decondensed, while the other (top center) is condensed; the dotted line shows the decondensed NOR region associated with its satellite. (**c**) Both NOR regions are decondensed; the fluorescent traces reveal the connection between the chromosomes and their satellites. (**d**) The NOR regions are decondensed; one NOR region is revealed by a fluorescent trail, and the other is indicated by a dotted line; the associated satellites appear as bright fluorescent signals but were not revealed by DAPI staining, as shown in the insert. Note in each metaphase the minor 35S rDNA site located in the pericentromeric region of a medium-size chromosome pair. Bar represents 10 µm.

To determine the number and location of the rDNA sites in *S. melongena*, *S. insanum* and *S*. *incanum*, we carried out FISH with 35S and 5S rDNA probes on all the accessions studied. Identical results were obtained in each eggplant cultivar, which allowed us to define a unique rDNA FISH mapping for this species. Two pairs of 35S sites (one major and one minor locus) and one pair of 5S rDNA sites were independently located on three different chromosome pairs (Figure 2a). The major 35S site was terminally located in the short arm of one large chromosome, in correspondence with the secondary constriction detected by the DAPI staining, while the minor site was mapped to the pericentromeric region of a medium-size chromosome.

The 5S rDNA sites were revealed by hybridization signals located in a chromosome pair of small size, apparently in the terminal position. However, the tendency of fluorescence to expand beyond the region labeled by the probe did not allow us to clearly distinguish between terminal or subterminal positions.

Metaphases displaying varying levels of NOR condensation were frequently observed, along with the simultaneous presence of condensed and decondensed NORs in the same cell. Condensed NORs were visualized as large fluorescent signals covering the secondary constrictions and satellites (Figure 2a). Conversely, in metaphases with highly elongated NORs, the satellites appeared as fluorescent signals, distant from the chromosome bodies that, however, were identified by a fluorescent band adjacent to the secondary constriction and, in some cases, by faint traces of fluorescence revealing the connection between the satellites and their respective chromosomes (Figure 2b–d). Furthermore, the hybridization signals highlighted satellites that remained undetected by DAPI staining. For instance, Figure 2d shows two prominent green signals corresponding to satellites not visible with DAPI. Metaphases with one or both decondensed NORs were observed indiscriminately in all cultivars and plants examined.

**Figure 3:**
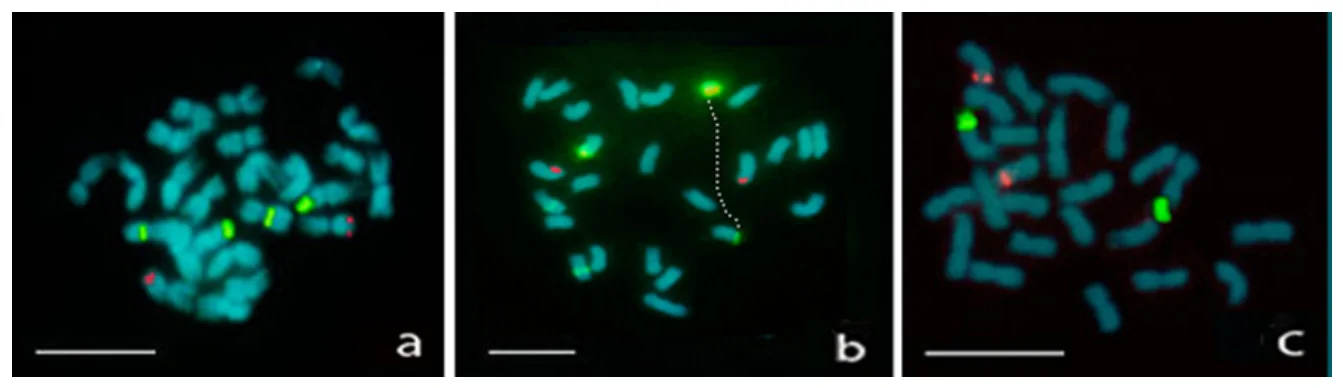
Fluorescent in situ hybridization with 35S (green) and 5S (red) rDNA probes in (**a**,**b**) *S. insanum* and (**c**) *S. incanum.* (**a**) Metaphase of *S. insanum* showing two large green signals corresponding to the condensed NOR regions. (**b**) One NOR is condensed, while the other is highly decondensed, the dotted line showing the association with its satellite. (**c**) Metaphase of *S*. *incanum* showing condensed NOR regions devoid of satellites. Note in *S. insanum* metaphases the 35S rDNA minor site located in the pericentromeric region of a pair of medium-size chromosomes; this minor site is missing in *S. incanum*. Bar represents 10 µm.

A similar rDNA FISH mapping was obtained in *S. insanum* (Figure 3a,b). Two large green signals corresponding to the major 35S rDNA sites and two small signals indicating the presence of the additional minor 35S sites were consistently observed. As in eggplant, secondary constrictions with varying levels of condensation were frequently observed in *S. insanum*. The number and position of the 5S rDNA sites, revealed by fluorescent red signals, reflect those detected in *S. melongena*.

In *S. incanum*, the 35S rDNA probe labeled only one pair of sites, which corresponded to the terminal NORs, visualized as poorly DAPI-stained regions (Figure 3c). The extension of the green signals confirmed that these regions are generally condensed. Highly stretched NORs, as well as additional minor sites, were never detected. Conversely, the number and location of the 5S rDNA sites were consistent with those observed in the other two species (Figure 3c).

### 3.3. s-GISH and Cross-GISH Experiments

**Figure 4:**
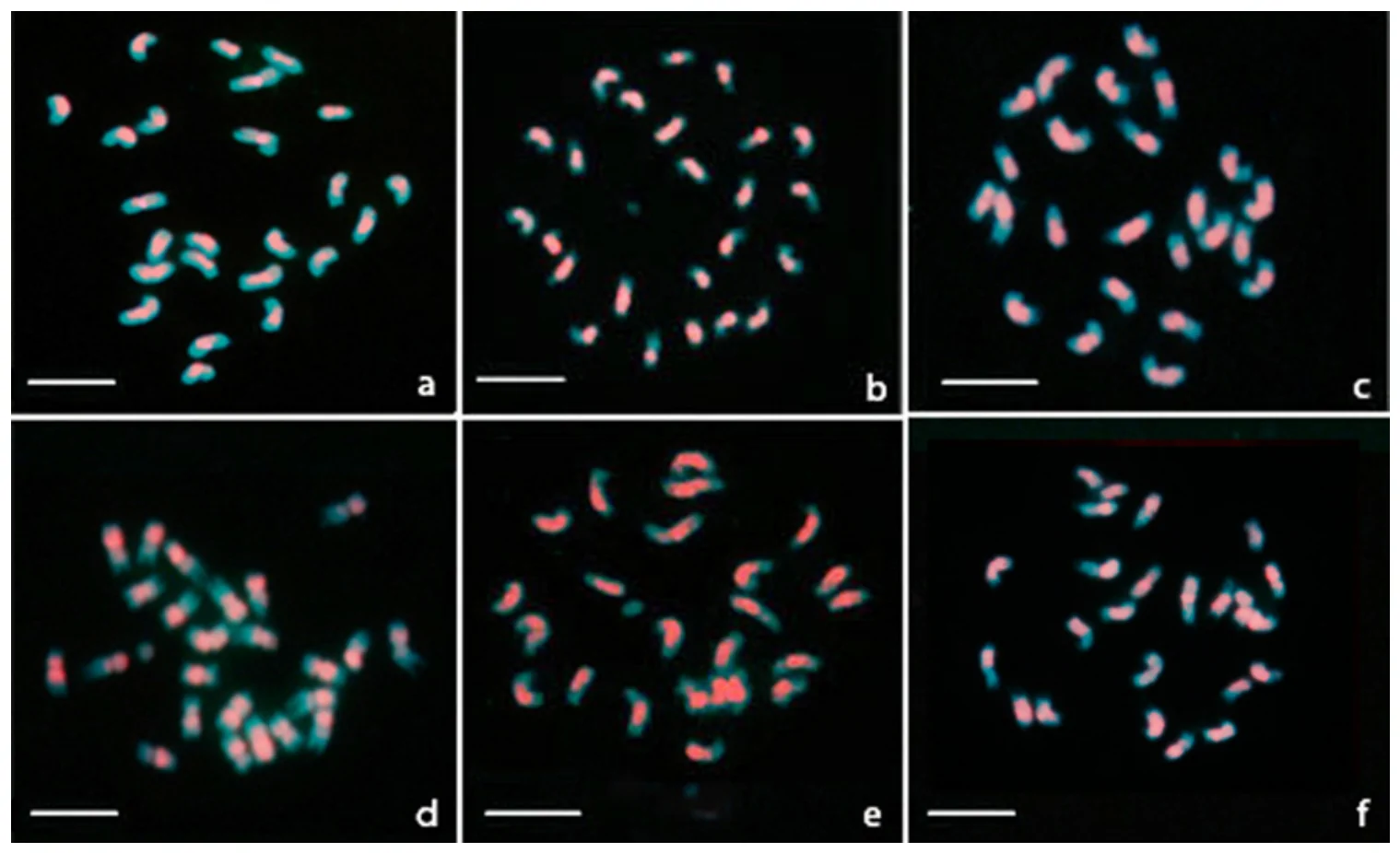
Chromosomal localization of repetitive DNA (red signal) in *Solanum* species by s-GISH and assessment of their genomic affinity by cross-GISH. Mitotic metaphases of (**a**) *S. melongena*, (**b**) *S. insanum* and (**c**) *S. incanum* after hybridization with their own genomic DNA. Note that, in each species, hybridization signals are concentrated in the centromeric and pericentromeric regions and proximal portions of the chromosome arms. Metaphases of *S. melongena* hybridized with the genomic DNA of (**d**) *S. insanum* and (**e**) *S. incanum*. (**f**) Metaphase of *S. insanum* hybridized with the genomic DNA of *S. incanum*. Note the substantial similarity between hybridization patterns generated in each species by s-GISH and cross-GISH experiments. Bar represents 10 µm.

The s-GISH technique was used to investigate the overall chromosome distribution of repetitive DNA sequences in the cultivated eggplant and its wild ancestors *S. insanum* and *S. incanum*. Almost identical s-GISH hybridization patterns were observed in the eggplant cultivars. The s-GISH pattern of the cultivar “White eggplant” is shown in Figure 4a, as an example. The genomic probe labeled all the chromosomes generating strong hybridization signals, which covered the centromeric and pericentromeric regions and the adjacent portions of both arms, while the distal parts of the arms, including the NOR regions and the satellites, remained unlabeled.

Similar s-GISH signal patterns were produced on the chromosomes of both *S. insanum* and *S. incanum* (Figure 4b,c). To evaluate the level of genome affinity among the three species, cross-GISH experiments were carried out by probing the genomic DNA of each species on metaphase chromosomes of the others. Metaphases of *S. melongena* hybridized with the genomic DNA of *S. insanum* and *S. incanum* are shown in Figure 4d and Figure 4e, respectively. A metaphase of *S. insanum* probed with the genomic DNA of *S. incanum* is shown in Figure 4f. Reciprocal GISH experiments produced similar results (images not shown). The fluorescent signal patterns generated by cross-GISH experiments confirmed the s-GISH results and demonstrated substantial genomic homology among *S. melongena*, *S. insanum* and *S. incanum*.

## 4. Discussion

Although applications of molecular cytogenetics in eggplant are relatively scarce and limited to specific experimental materials, such as inbred lines, a few cultivars, and samples of unknown origin [18,26,34], they have provided some contributions to chromosomal identification and karyotyping [18,34]. Nevertheless, the full potential of in situ hybridization techniques in this species remains unexploited. For instance, the GISH procedure, a highly effective method for examining repetitive DNA organization in plant genomes, has never been used. Furthermore, the almost total absence of cytogenetic studies in *S. insanum* and *S. incanum* is remarkable, despite their central role in the origin of the cultivated eggplant. To address these gaps, we integrated chromosomal morphology, rDNA FISH mapping, and GISH patterns to characterize the chromosome complements of *S. melongena*, *S. insanum*, and *S. incanum* and to elucidate their critical evolutionary similarities and differences. FISH experiments, carried out here for the first time on *S. insanum* and *S. incanum*, have provided essential insights into their chromosome structure. Additionally, the rDNA FISH mapping in eggplant integrated previous fragmentary data [18,34,35] by the simultaneous localization of the 5S and major and minor 35S sites, which were clearly and unequivocally identified across all examined cultivars. The 35S rDNA minor site was also consistently observed in *S. insanum* but not in *S. incanum*. The absence of the secondary constriction at this site suggests that these sequences are transcriptionally inactive and, therefore, do not function as NORs. Analysis of the nucleolar chromosomes highlighted further similarities between *S. melongena* and *S. insanum*, notably, the presence of prominent satellites and secondary constrictions often found in a high state of decondensation. The localization of the fluorescent signals in decondensed secondary constrictions showed that the 35S rDNA site spans the entire satellite, the secondary constriction, and the adjacent region of the short arm. Conversely, the 35S site of *S. incanum* is restricted to the secondary constriction and the adjacent region of the short arm, reflecting the absence of the large satellite found in the other two species. The different NOR condensation/decondensation level appears to be a real trait characterizing the three species, as a strictly standardized protocol for chromosome preparations was applied. The 35S rDNA FISH signals detected in eggplant and *S. insanum* compared to *S. incanum* suggest that transposition and amplification mechanisms occurred during the evolution of *S. insanum* from *S. incanum.* This pattern was then conserved during eggplant domestication.

Secondary constrictions and the adjacent portions of chromosome arms are typically DAPI-negative. This feature has been documented in several plant groups, including various Solanaceae species [36,37,38], woody angiosperms such as *Castanea* spp. [39], and pteridophytes like *Selaginella* [40]. We found that the absence of DAPI staining extends to the entire satellite region, which often remained invisible in *S. melongena* and *S. insanum*. Similar results were obtained by Bhowmick et al. [26], who first analyzed the *S. insanum* chromosomes. Secondary constrictions are generally surrounded by heterochromatin that may be characterized by base-specific fluorochromes such as chromomycin A (CMA) and DAPI that exhibit preferential staining for GC- and AT-rich DNA sequences, respectively [40,41]. Our findings show that the proximal and distal regions surrounding the secondary constrictions, as well as the satellites, consist of heterochromatin with a high GC content.

The success of GISH techniques in cytogenetic and evolutionary studies in plants is well documented [42,43,44]. The hybridization of genomic DNA to chromosomal DNA depends on the homology of repetitive sequences, which are typically divided into interspersed repeats and tandem repeats. Among the latter, satDNA constitutes the most abundant fraction. SatDNA sequences are primarily localized to the heterochromatic regions of centromeres and subtelomeres, thereby contributing to the structural integrity of these vital chromosomal regions. Despite this, satDNA exhibits a highly dynamic nature, which leads to rapid changes in sequence composition and array size over short evolutionary timescales [45]. The homology of satDNA repeats is, therefore, a valuable indicator for assessing genetic similarity and phylogenetic relationships among related taxa.

In this study, the GISH technique was employed to gain insight into the chromosome organization of the three species and to evaluate their genomic affinity. The s-GISH results highlighted a common pattern of satDNA distribution across the three *Solanum* species. Signals were concentrated in the centromeric and pericentromeric regions, which indicates a predominant distribution of genes in the distal portions of the chromosomes. These data agree with the analysis of gene distribution in eggplant by Wei et al. [18], who described a relatively high gene density in the terminal regions of the chromosomes.

Overall, the results revealed substantial similarity among the three species examined. However, they highlighted specific cytogenetic features shared by the cultivated eggplant and its direct ancestor, which clearly distinguish them from *S. incanum*. These findings align with the known evolutionary history of these species and confirm the inclusion of *S. insanum* and *S. incanum* in the primary eggplant gene pool [46].

## 5. Conclusions

In this study, we performed a comparative cytogenetic characterization of the cultivated eggplant and its wild relatives, *S. insanum* and *S. incanum*. The combination of karyotypic analysis, FISH, and GISH techniques significantly advanced our understanding of the chromosomal organization of these species, highlighting both their shared and distinguishing characteristics.

Karyotype analysis and rDNA FISH mapping revealed marked similarities between *S. melongena* and *S. insanum*. By contrast, *S. incanum* was distinguished by its nucleolar chromosome structure and the absence of a minor 35S rDNA site, which was clearly observed in the other two species.

The satDNA chromosomal distribution analyzed by the s-GISH technique revealed similar patterns in eggplant, *S. insanum* and *S. incanum*, which suggests that the amount of repetitive DNA in these species has been substantially conserved throughout their evolution. Moreover, the hybridization signals generated by cross-GISH experiments reflected the s-GISH patterns, definitively confirming the genomic homology among the three species.

Studies on the domestication of the eggplant have established *S. insanum* and *S. incanum* as the direct ancestor and closest wild relative, respectively. Our results complement previous research by offering cytogenetic evidence for the evolutionary relationships among these species.

## Figures and Tables

**Table 1:** List of the cultivars and accessions of the analyzed *Solanum* species.

Species	Cultivar/Accession	
*Solanum melongena* L.	White	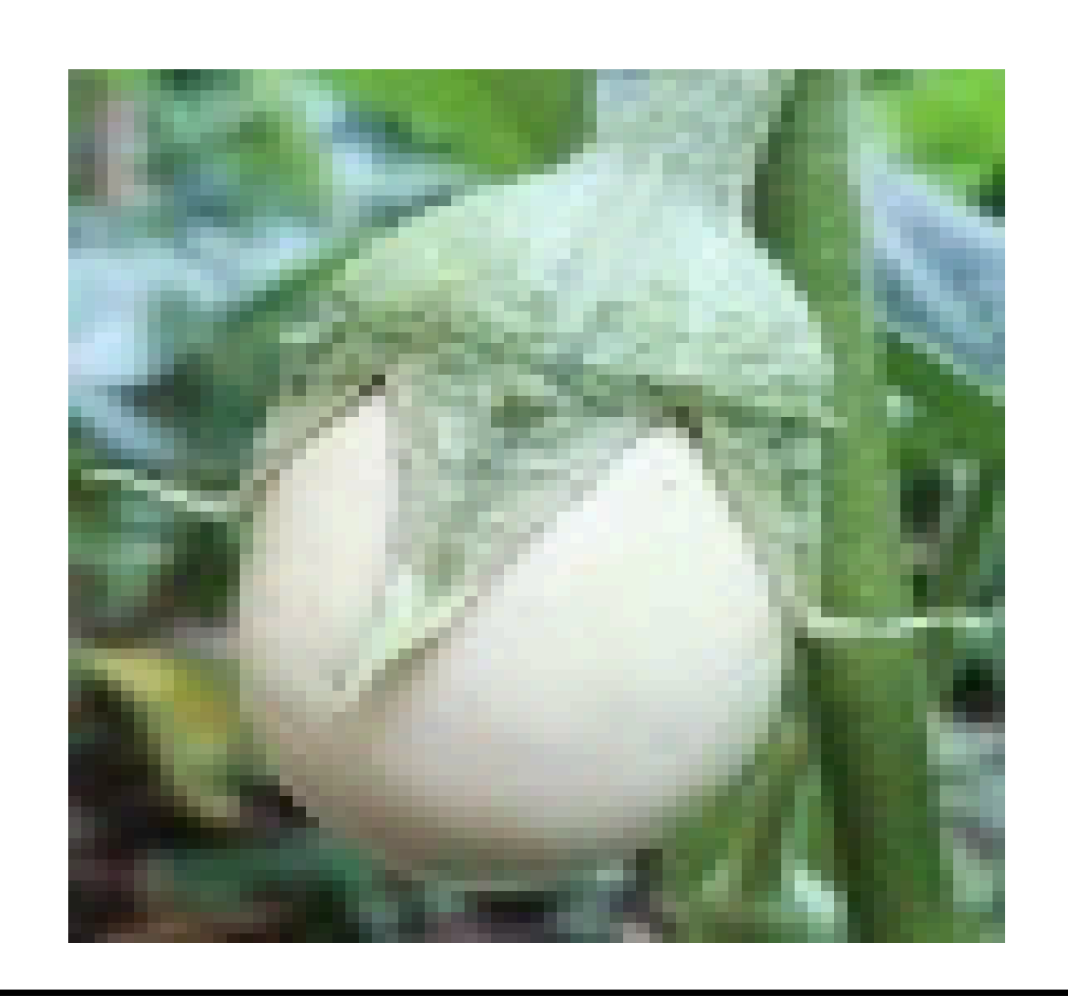
	Globe purple	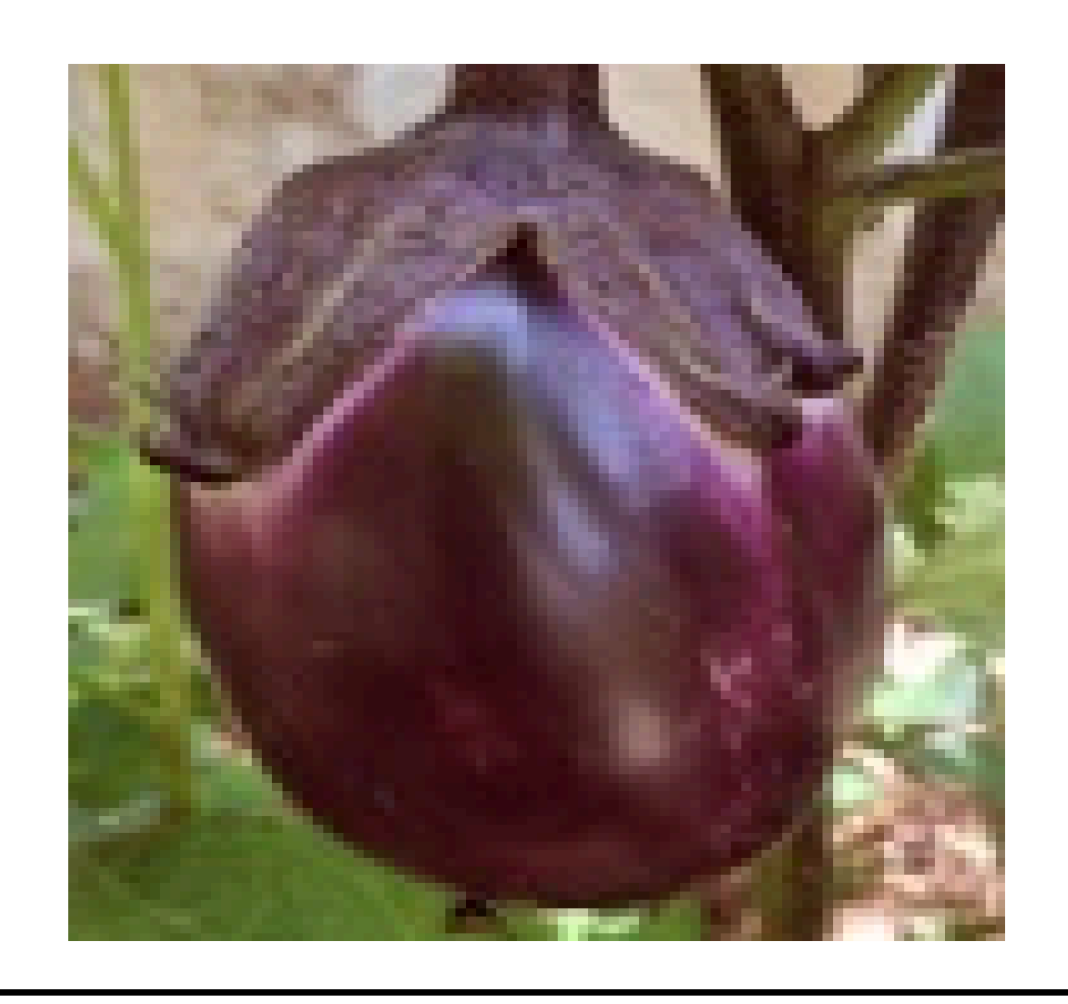
	Black beauty	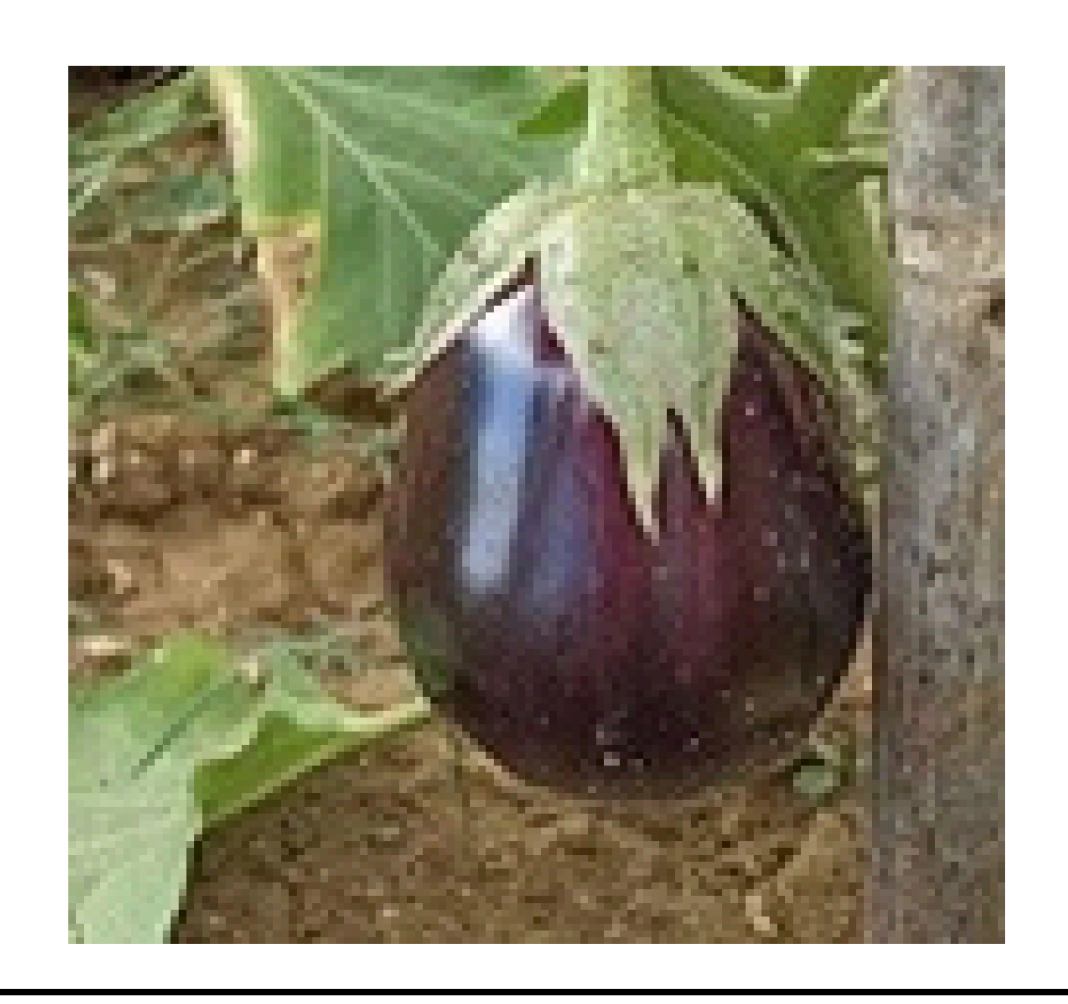
	Stripped	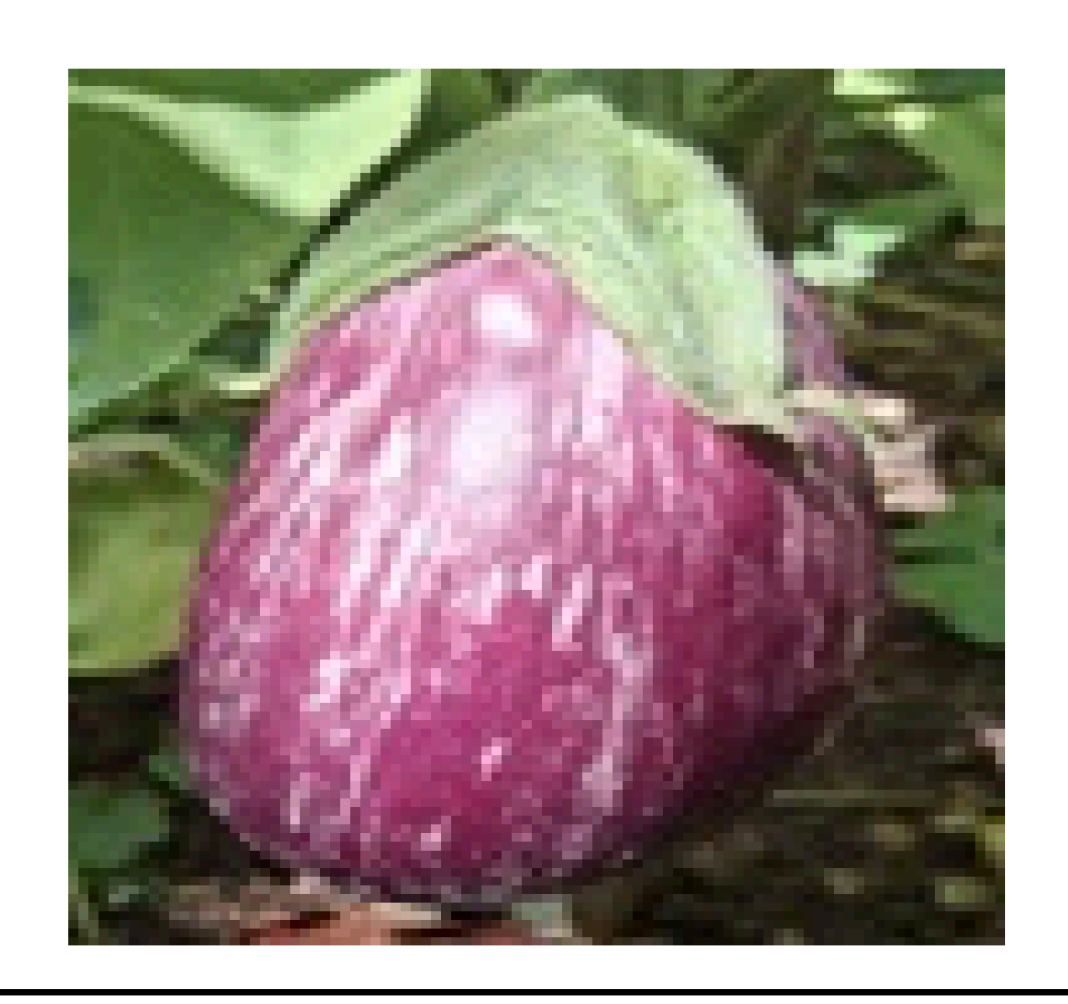
	Round purple	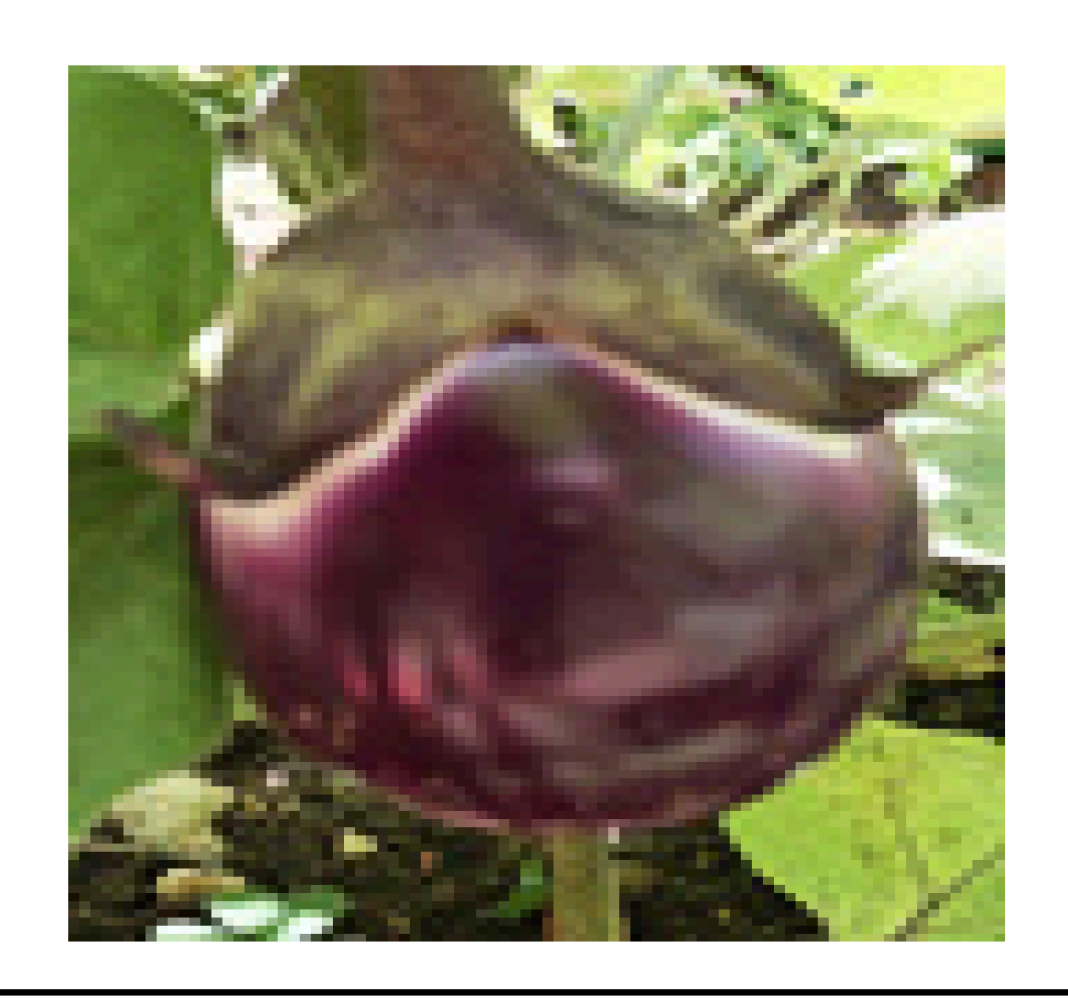
	Round black	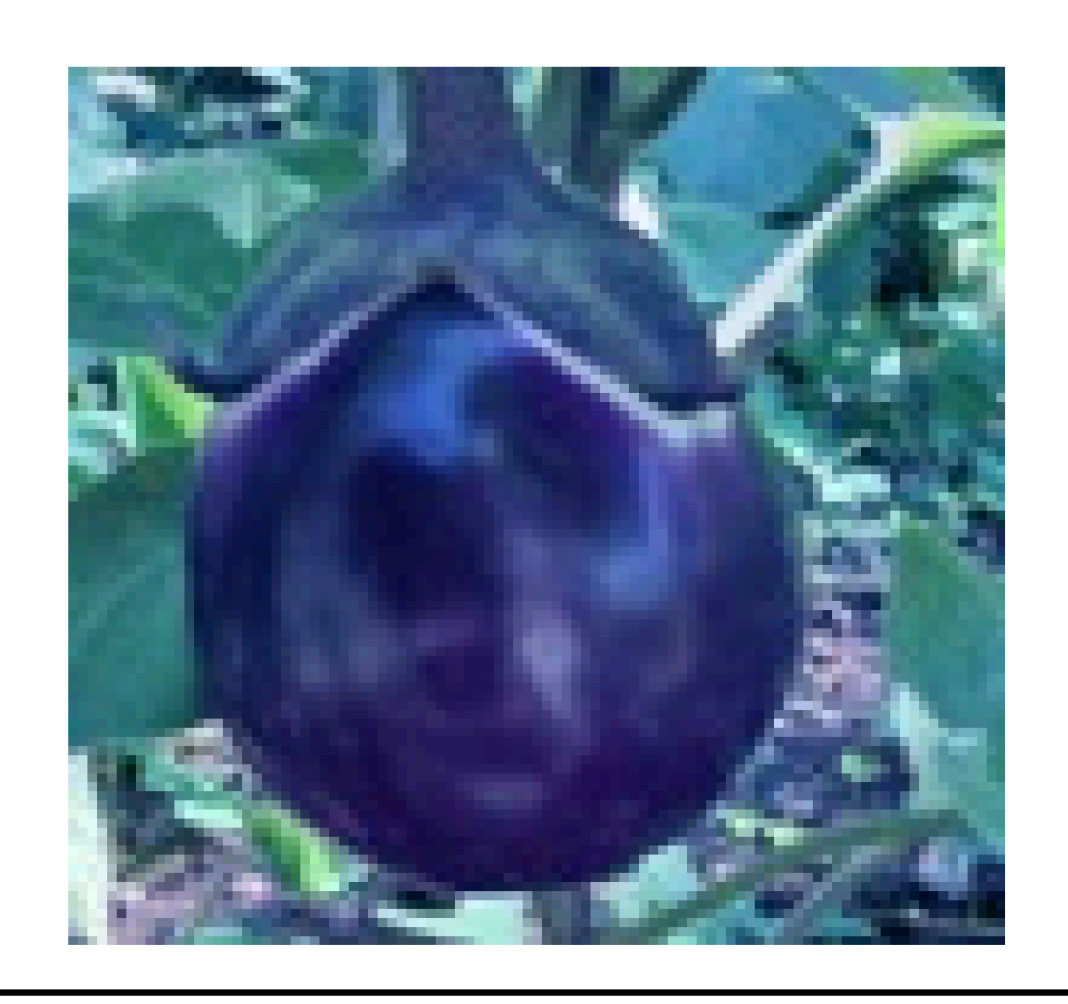
	White pink	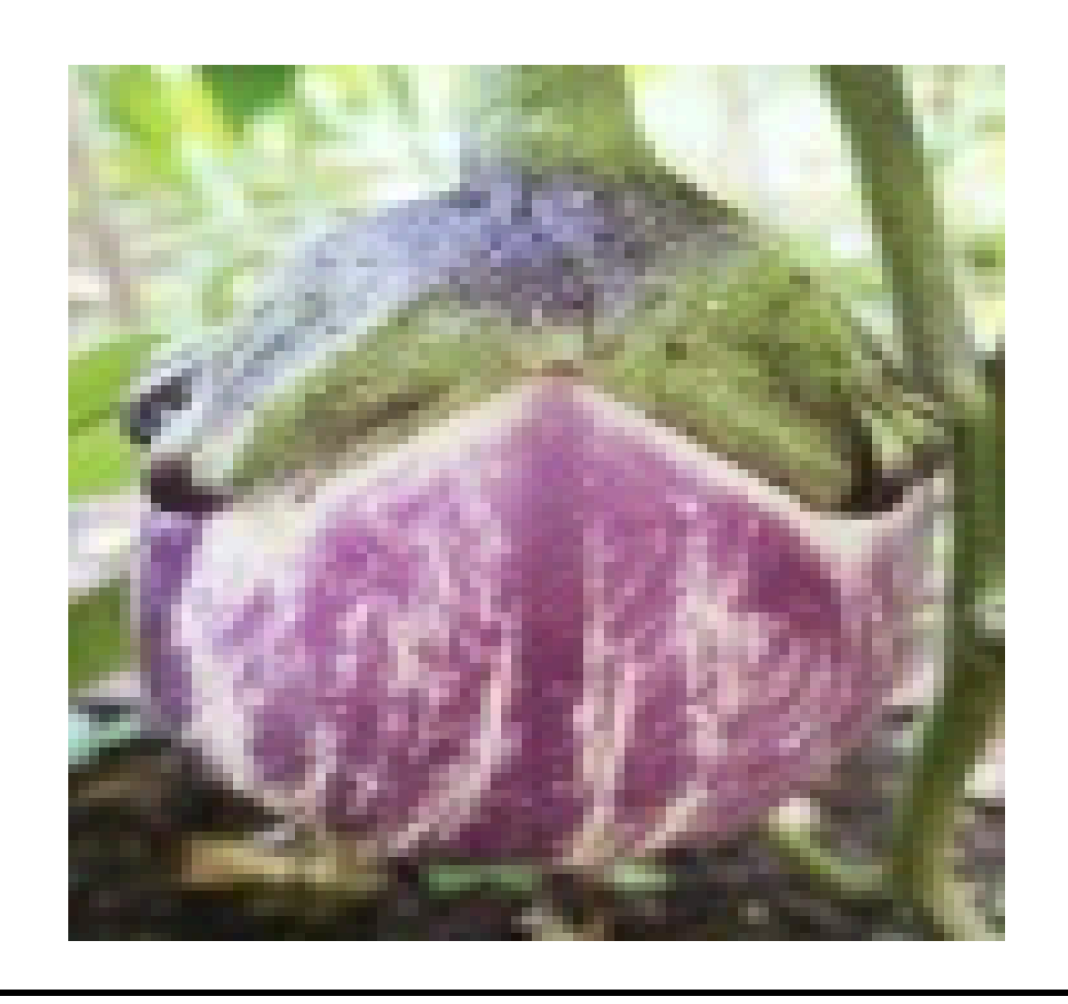
*Solanum insanum* L.	INS1	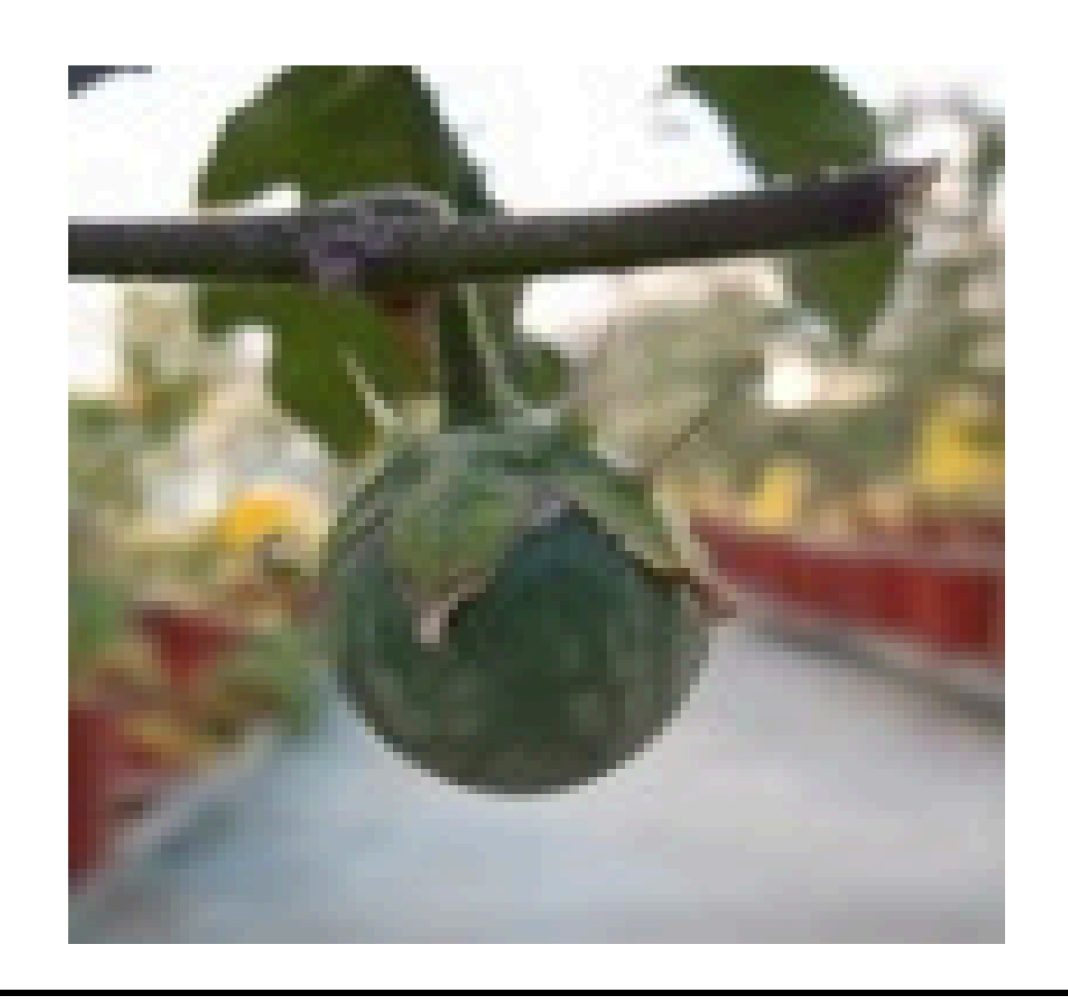
*Solanum incanum* L.	MM577	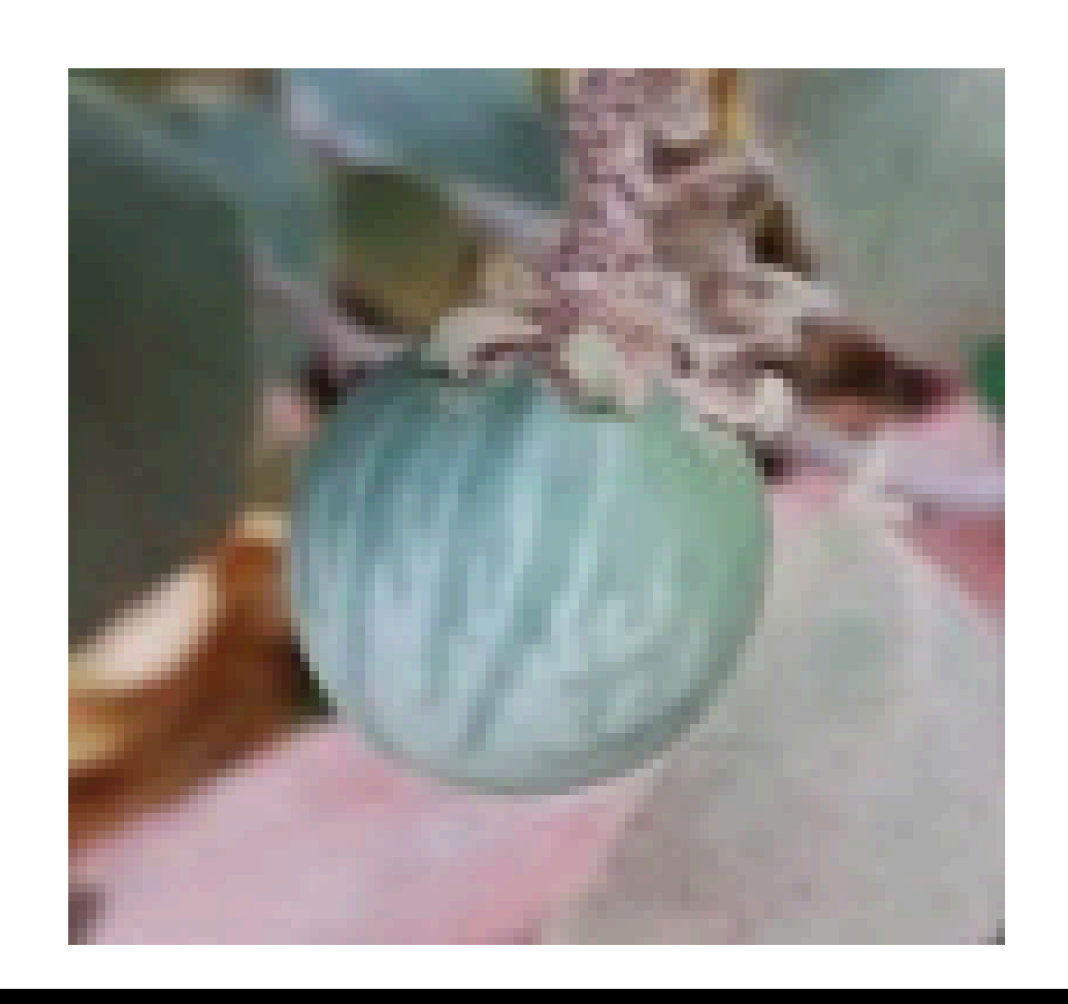

## Data Availability

The original contributions presented in this study are included in the article. Further inquiries can be directed to the corresponding author.
